# Comparing Artificial Neural Networks, General Linear Models and Support Vector Machines in Building Predictive Models for Small Interfering RNAs

**DOI:** 10.1371/journal.pone.0007522

**Published:** 2009-10-22

**Authors:** Kyle A. McQuisten, Andrew S. Peek

**Affiliations:** Department of Bioinformatics, Integrated DNA Technologies, Inc., Coralville, Iowa, United States of America; Universität Heidelberg, Germany

## Abstract

**Background:**

Exogenous short interfering RNAs (siRNAs) induce a gene knockdown effect in cells by interacting with naturally occurring RNA processing machinery. However not all siRNAs induce this effect equally. Several heterogeneous kinds of machine learning techniques and feature sets have been applied to modeling siRNAs and their abilities to induce knockdown. There is some growing agreement to which techniques produce maximally predictive models and yet there is little consensus for methods to compare among predictive models. Also, there are few comparative studies that address what the effect of choosing learning technique, feature set or cross validation approach has on finding and discriminating among predictive models.

**Principal Findings:**

Three learning techniques were used to develop predictive models for effective siRNA sequences including Artificial Neural Networks (ANNs), General Linear Models (GLMs) and Support Vector Machines (SVMs). Five feature mapping methods were also used to generate models of siRNA activities. The 2 factors of learning technique and feature mapping were evaluated by complete 3×5 factorial ANOVA. Overall, both learning techniques and feature mapping contributed significantly to the observed variance in predictive models, but to differing degrees for precision and accuracy as well as across different kinds and levels of model cross-validation.

**Conclusions:**

The methods presented here provide a robust statistical framework to compare among models developed under distinct learning techniques and feature sets for siRNAs. Further comparisons among current or future modeling approaches should apply these or other suitable statistically equivalent methods to critically evaluate the performance of proposed models. ANN and GLM techniques tend to be more sensitive to the inclusion of noisy features, but the SVM technique is more robust under large numbers of features for measures of model precision and accuracy. Features found to result in maximally predictive models are not consistent across learning techniques, suggesting care should be taken in the interpretation of feature relevance. In the models developed here, there are statistically differentiable combinations of learning techniques and feature mapping methods where the SVM technique under a specific combination of features significantly outperforms all the best combinations of features within the ANN and GLM techniques.

## Introduction

Exogenous small interfering RNAs (siRNAs) can be introduced into cells, enter endogenous pathways and reduce the amount of their target RNA [1]. However, not all siRNAs perform this knockdown function with equal efficacy [2]–[7]. Many studies have developed models for siRNA efficacy and a heterogeneous group of learning techniques have been used in the development of predictive siRNA models, Table 1 [8]–[38]. In addition to the various learning techniques, the number of feature mapping methods and the number of datasets that have been used to develop models for siRNAs are also large and heterogeneous, Table 1. The thirty works enumerated in Table 1 individually provide more details about the specific approaches being used to computationally model siRNAs, as well as many other un-cited works that have developed more precise biochemical understandings of the various siRNA and miRNAs mechanisms. However together, these works provide a glimpse as to the heterogeneity in methodologies that have been taken, and while each approach is certainly valid, modest efforts have been made to synthesize across approaches to ascertain what commonalities exist and where enhancements in comparisons can be made among approaches.

**Table 1:** Computational systems used in developing models for predicting effective RNAi.

#	Technique(s)	class/reg	siRNA data set	Total Features	Reference(s)
1	Rule	classification	180–19mers	8	[8]
2	Rule	classification	62–19mers	4	[9]
3	Rule	classification	46–19mers-train, 34–19mers-test	9	[10]
4	Rule	classification	148–19mers	18	[11]
5	Rule	classification	249–19mers	12	[12]
6	Rule	classification	23–19mers	2	[13]
7	GPBoost, SVM	class/reg	204–19mers	?	[14]
8	GPBoost, SVM	regression	581–19mers	?	[15]
9	DT	class/reg	398–19mers	11	[16]
10	Rule	classification	composite	8	[17]
11	ANN	regression	2431–21mers	84	[18], [19]
12	ANN	classification	180–19mers	6	[20]
13	Rule, DT	classification	601–19mers	55	[21]
14	GSK SVM	classification	94–19mers	84	[22]
15	Rule DT, SVM	classification	33–21mers	4	[23]
16	SVM	classification	2431–21mers, 581–19mers	84+15+20	[24]
17	ANN	regression	581–19mers-train, 2431–21mers-test	200	[25]
18	linear	regression	526–19mers	84	[26]
19	linear	regression	2431–21mers, 653–19mers	84+84	[27]
20	DRM	classification	3277	276-initial 21-final	[28]
21	Rule	classification	420 and 1220	6+4+16+64	[29]
22	SVM	class/reg	2252–21mers, 240–19mers	572	[30]
23	linear	regression	2431–21mers	84+	[31]
24	SVM	regression	2431–21mers, 579–19mers	1566	[32]
25	Rule, DT, GPBoost, ANN, linear	class/reg	2431–21mers, 601–19mers, 238–19mers, 67–19mers	84+84, 22-final	[33]
26	SVM	classification	2431–21mers, 653–19mers	28	[34]
27	Rule, SVM, RFR	regression	3589	41	[35]
28	linear	regression	702–19mers	76+3	[36]
29	Rule HS	classification	474 subset of 2433–21mers, 99 subset of 294–21mers, 360 21–mers	4	[37]
30	Rule DT	classification	62 21-mers	8	[38]

GPBoost: Genetic Programming and Boosting.

SVM: Support Vector Machine.

DT: Decision Tree.

ANN: Artificial Neural Network.

GSK: General String Kernel.

DRM: Disjunctive Rule Merging.

RFR: Random Forest Regression.

HS: Hierarchical Sorting.

Statistical learning techniques have fallen into two broad categories. The first group of learning techniques involves the development of models that classify siRNAs into discrete groups of more effective and less effective, based on their properties or features. The second group of learning techniques involves the development of a regression model that predicts a siRNA's effectiveness from a continuous distribution, also based on the siRNA properties, or feature set. In this second group, three common learning techniques that have been used to develop predictive regression models are Artificial Neural Networks (ANNs), General Linear Models (GLMs) and Support Vector Machines (SVMs). Here we intend to more closely investigate what the choice of feature set, learning technique, measure of model precision or accuracy and statistical test has on making conclusions about predictive models.

A model is comprised of several components; minimally a model involves a learning technique, a set of features on which to learn and then a dataset which contains the features and the outcome (or outcomes) of interest. Development of models that predict the effectiveness of small interfering RNAs (siRNAs) are useful for several reasons. First, and perhaps most trivially, models are used to develop ever more predictively functional schemes. Second, models can be used to better understand the system under study. As a crude sketch of a complex system, the model encapsulates features that associate with effective or ineffective siRNAs and can lead to insights into the structures, functions and mechanisms of siRNAs. Third, the model building procedures can be compared to determine what combinations of learning techniques and feature mapping methods that are able to generate significantly effective models on the data under study. Namely, models are simply formalized hypotheses and as such models can be compared in their abilities to explain and predict with associated measures of precision and accuracy.

Two general criteria are used in the evaluation of a model's ability to predict data not seen in model training: model precision and model accuracy. Model precision is based on the ability to fit a relationship between predicted and empirically observed activities (namely the Pearson correlation or *R* fit of the model between predicted and observed). Model accuracy is based on the ability to fit a relationship between predicted and observed that minimizes the residuals between the predicted and empirically observed activities (namely the Mean Squared Error or *MSE* of the model). Previous studies investigating siRNA activities have generally not discriminated between machine learning techniques and feature mapping methods. No general comparisons have been made to systematically understand the performance of identical features with different learning techniques or identical learning techniques with different features for siRNAs. Here the intention is to more closely investigate the effect of choosing ANN, GLM and SVM learning techniques and feature mapping methods in the development of predictive siRNA regression models from estimates of their precision and accuracy.

## Results

### I. Individual learning techniques and feature mapping methods

#### Ia. training and testing models on the entire dataset

The three learning techniques of ANN, GLM and SVM were used to develop predictive models for the same dataset of 2431 siRNAs across the 5 feature mapping methods of 1) Position Specific Base Composition (PSBC), 2) Thermodynamics (THER), 3) N-Grams of length 2 through 5 (NG25), 4) Guide Strand Structural Features (GSSF) and 5) Guide Strand Secondary Structure (GSSS). Both training the models and then testing their precision on the entire dataset resulted in models with correlations (*R*) between predicted and observed activities that ranged from 0.198 to 0.897 (GLM-GSSF and SVM-NG25, respectively), Table 2. Similarly the entire dataset was used to determine model accuracies by both training and then testing the model to determine the Mean Squared Errors (*MSE*) between predicted and observed activities that ranged from 0.009 to 0.936 (SVM-NG25 and GLM-NG25, respectively), Table 2.

**Table 2:** Model performance by learning technique and feature mapping method for correlations and mean squared error on the entire dataset and by 10-fold cross validation.

		ANN				GLM				SVM			
		Train on 2431	Test on 2431	10-fold	Cross validation	Train on 2431	Test on 2431	10-fold	Cross validation	Train on 2431	Test on 2431	10-fold	Cross validation
Mapping method	Number Features	*R*	*MSE*	*R*	*MSE*	*R*	*MSE*	*R*	*MSE*	*R*	*MSE*	*R*	*MSE*
PSBC	84	0.658	0.023	0.636	**0.025**	0.631	0.029	**0.607**	**0.031**	0.764	0.017	0.643	0.024
THER	23	0.562	0.028	0.567	0.029	0.514	0.840	0.511	0.844	0.722	0.019	0.579	0.027
NG25	1360	0.871	0.015	0.464	0.049	0.450	0.936	0.357	0.929	0.897	0.009	0.509	0.030
GSSF	32	0.316	0.036	0.278	0.038	0.198	0.072	0.152	0.115	0.232	0.039	0.215	0.039
GSSS	23	0.301	0.037	0.279	0.038	0.207	0.091	0.201	0.091	0.339	0.036	0.271	0.038
P+13	168	0.703	0.021	**0.660**	0.027	0.500	0.252	0.474	0.257	0.779	0.010	0.681	0.022
P+25	1444	0.898	0.012	0.572	0.047	0.513	1.144	0.439	1.109	0.931	0.006	**0.711**	**0.020**
ALL	1522	0.430	0.136	0.524	0.055	0.509	2.605	0.444	2.529	0.934	0.006	0.644	0.025

Learning Techniques: Artificial Neural Network (ANN), General Linear Model (GLM), Support Vector Machine (SVM).

Mapping methods: Position Specific Base Composition (PSBC), Thermodynamic (THER), N-Grams of length 2 though 5 (NG25), Guide Strand Structure Features (GSSF), Guide Strand Secondary Structure (GSSS), Positions specific base compositions plus N-Grams of length 1 through 3 (P+13), Positions specific base compositions plus N-Grams of length 2 through 5 (P+25) the combination of each of the methods PSBC, THER, NG25, GSSF and GSSS (ALL).

*R*  =  Pearson correlation coefficient, of model predicted activities to observed activities.

*MSE*  =  Mean Squared Error of model predicted activities to observed activities.

column maxima for *R* and minima for *MSE* are in **bold** for 10-fold cross validations, same values bolded in Table 7.

#### Ib. 10-fold cross-validation

Since training and testing a model on the same dataset is not a realistic measure of model performance 10-fold stratified cross validation was used. Briefly, cross validation involves partitioning the dataset into M subsets, so that each subset contains a maximal distribution of the siRNA activities, and the model was trained on M-1 of these and then tested on the remaining hold-out subset. This is repeated for each of the partitions to generate M (mostly) independent estimates of model performance. Using 10-fold stratified cross validation resulted in models with correlations (*R*) between predicted and observed activities that ranged from 0.152 to 0.643 (GLM-GSSF and SVM-PSBC, respectively), Table 2. Similarly the 10-fold cross validation resulted in models with the Mean Squared Errors (*MSE*) between predicted and observed activities that ranged from 0.024 to 0.929 (SVM-PSBC and GLM-NG25, respectively), Table 2. In general 10-fold cross validation model values are lower for precision and accuracy (decreased *R*, increased *MSE*) than models trained and tested on the entire dataset due to the overfitting problem.

### II. 3×5 ANOVA on *R* and *MSE* from 10-fold cross validation replicates

To more completely understand the non-obvious contributions of both the learning technique and feature mapping methods on determining model precision and accuracy the results of the ten individual cross validations were treated as repeated measures within a complete factorial analysis of variance (ANOVA). For determining the sources of variation in measures of model precision, the variance in model correlations (*R*) were evaluated under 4 ANOVA model assumptions, Table 3. The first model M*_R_*_1_ contained marginally significant evidence for the variance in *R* being influenced by choice of learning technique alone. The second model M*_R_*_2_ contained evidence for highly significant contribution to the variance in *R* by choice of feature mapping method alone. The model M*_R_*_3_ containing both learning techniques and mapping methods, but without interactions between techniques and features, contained a significantly better fit to either the M*_R_*_1_ or M*_R_*_2_ model that contained only learning techniques alone or mapping methods alone, Table 4. Finally the model M*_R_*_4_, that contained interaction terms between techniques and methods, had a marginally significantly better fit than the model M*_R_*_3_, without interaction terms, Table 4.

**Table 3:** Individual model ANOVA on correlation (*R*) cross validation replicates.

Mdl	Model formula	*R^2^*	*R.S.S.*	*d.f.*	*F*	*P*
M*_R_*_1_	*R* = technique + error	0.02958	4.5157	2, 147	3.27	0.041
M*_R_*_2_	*R* = method + error	0.8314	0.7741	4, 145	184.6	<2.2×10^−16^
M*_R_*_3_	*R* = technique + method + error	0.8734	0.5731	6, 143	172.3	<2.2×10^−16^
M*_R_*_4_	*R* = technique + method + (technique×method) + error	0.8822	0.5033	14, 135	80.73	<2.2×10^−16^

**Table 4:** Model comparisons by ANOVA for *R*.

Mdl*_A_*	Mdl*_B_*	*R.S.S._A_*	*R.S.S._B_*	*d.f._A_*	*d.f._B_*	*d.f.*	*F*	*P*
M*_R_*_1_	M*_R_*_3_	4.5157	0.5731	147	143	4	245.93	<2.2×10^−16^
M*_R_*_2_	M*_R_*_3_	0.7741	0.5731	145	143	2	25.069	4.646×10^−10^
M*_R_*_3_	M*_R_*_4_	0.5731	0.5033	143	135	8	2.3414	0.02181

A similar procedure was used for evaluating the sources of variation for *MSE* estimates between the learning techniques and feature mapping methods. The first *MSE* model M*_MSE_*_1_ contained highly significant evidence for the variance in *MSE* being influenced by choice of learning technique alone, Table 5. The second model M*_MSE_*_2_ contained evidence for significant contribution to the variance in *MSE* by choice of feature mapping method, Table 5. The model M*_MSE_*_3_ containing both learning techniques and mapping methods, but without interactions between techniques and features M*_MSE_*_4_, contained a significantly better fit to either the M*_MSE_*_1_ or M*_MSE_*_2_ models, Table 6. Finally the model M*_MSE_*_4_, that contained interaction terms between techniques and methods, had a highly significantly better fit than the model M*_MSE_*_3_, without interaction terms, Table 6. In summary, both learning techniques and mapping methods contribute to the source of variation in measures of model precision (*R*) and accuracy (*MSE*), but contribute to various degrees to each.

**Table 5:** Individual model ANOVA on Mean Squared Error (*MSE*) cross validation replicates.

Mdl	Model formula	*R^2^*	*R.S.S.*	*d.f.*	*F*	*P*
M*_MSE_*_1_	*MSE* = technique + error	0.3535	7.9759	2, 147	41.73	4.442×10^−15^
M*_MSE_*_2_	*MSE* = method + error	0.1904	9.8519	4, 145	9.759	5.107×10^−7^
M*_MSE_*_3_	*MSE* = technique + method + error	0.5564	5.3238	6, 143	32.14	<2.2×10^−16^
M*_MSE_*_4_	*MSE* = technique + method + (technique×method) + error	0.9931	0.0778	14, 135	1540	<2.2×10^−16^

**Table 6:** Model comparisons by ANOVA for *MSE*.

Mdl*_A_*	Mdl*_B_*	*R.S.S._A_*	*R.S.S._B_*	*d.f._A_*	*d.f._B_*	*d.f.*	*F*	*P*
M*_MSE_*_1_	M*_MSE_*_3_	7.9759	5.3238	147	143	4	17.81	6.937×10^−12^
M*_MSE_*_2_	M*_MSE_*_3_	9.8519	5.3238	145	143	2	60.815	<2.2×10^−16^
M*_MSE_*_3_	M*_MSE_*_4_	5.3238	0.0778	143	135	8	1138	<2.2×10^−16^

### III. Feature set selection for maximizing precision and accuracy

Due to the interaction between learning technique and feature mapping method in determining model accuracy a brute force survey approach was used to find both precise and accurate models and limited to the 3 learning techniques and 5 feature mapping methods. Feature mapping methods were evaluated by combining and filtering to find combinations of features that maximized *R* and minimized *MSE* under the 3 learning techniques. The 5 feature mapping methods PSBC, THER, NG25, GSSF and GSSS were evaluated in all 31 combinations then filtered across 9 increasingly stringent levels of feature inclusion and finally measured for *R* and *MSE* across the 3 learning techniques by 10-fold cross validation. Combined there were a total of 837 models evaluated for *R* and *MSE* by 10-fold cross validation. The ANN learning technique had a maximal value of *R* = 0.660 with the P+13 feature mapping method (the method PSBC combined with N-Grams of length 1 through 3) and minimal values of *MSE*  = 0.025 with the PSBC method. The GLM learning technique had a maximal value of *R* = 0.607 and minimal values of *MSE*  = 0.031 both with the PSBC method. The SVM learning technique had a maximal value of *R* = 0.711 and minimal values of *MSE*  = 0.020 both with the P+25 mapping method (the method PSBC combined with N-Grams of length 2 through 5), Table 2.

### IV. Comparisons among models

#### IVa. within learning technique, between feature mapping method comparisons

**Figure 1:**
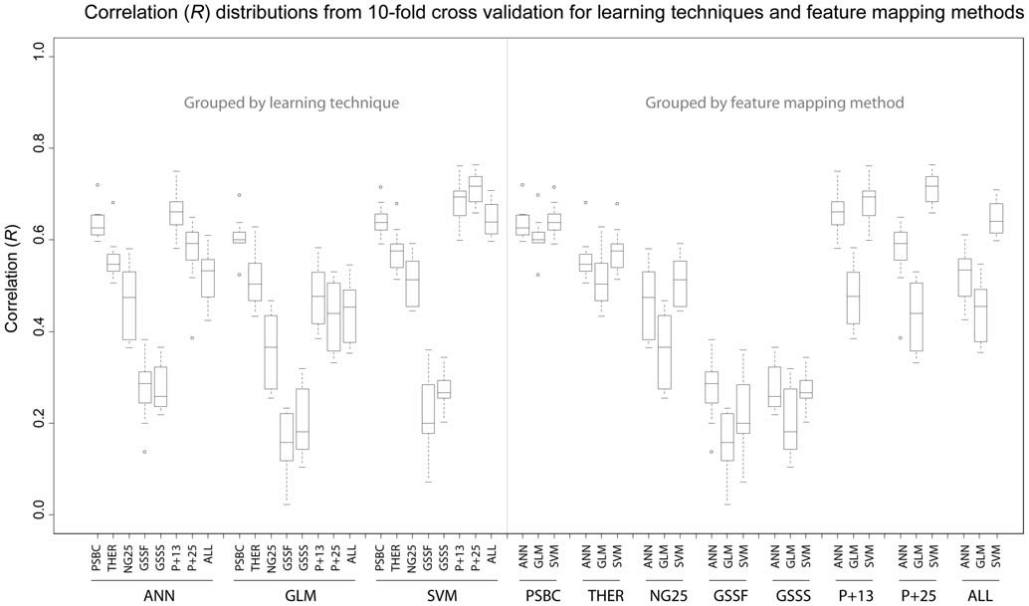
Box-and-whisker diagrams for the cross validation estimates of model precision performance, or Pearson correlation (*R*). Boxes encompass the first to third quartile of the distribution. The medians of the distributions are given as horizontal lines within the boxes. Whiskers encompass the 5% to 95% confidence regions of the distribution. Statistical outliers are shown as open circles. The left side of the diagram groups the model precision estimates by machine learning technique. The right side of the diagram groups the model precision estimates by feature mapping method.

Within the ANN learning technique, the feature mapping method that produced the model with the highest precision is the P+13 method, with a mean *R* = 0.660 under 10-fold cross validation. The distribution ranges of the 10-fold cross validation estimates of *R* are presented in Figure 1, first grouped by learning technique, then by feature mapping method. It is apparent in Figure 1 that the variances of the best performing method, P+13, overlaps with the next most precise method, PSBC (*R* = 0.636). Determining whether *R* = 0.660 is significantly greater than *R* = 0.636 is a matter of performing a 2 population *t*-test for the comparisons of means between the 10-fold cross validation estimates of the model *R*. In this case the H_0_: x_1_ = x_2_ is unable to be rejected *P* = 2.26E-01. However, in the case of the comparisons between the method of P+13 and other methods within the ANN technique the null hypothesis of equality of means of *R*, are able to be rejected with various degrees of statistical confidence, Table 7.

**Table 7:** Comparison among learning technique and mapping method for building significantly dissimilar models by 10-fold cross validation.

TEC		ANN							
	MET	PSBC	THER	NG25	GSSF	GSSS	P+13	P+25	ALL
	PSBC	0.636 **0.025**	8.89E-04**	2.26E-05**	2.20E-09**	1.41E-11**	2.26E-01	3.02E-02*	1.21E-04**
	THER	2.76E-03*	0.567 0.029	5.70E-03*	2.31E-08**	7.02E-10**	1.48E-04**	6.00E-01	2.02E-01
	NG25	3.30E-05**	1.37E-04**	0.464 0.049	2.44E-05**	1.03E-05**	5.23E-06**	5.13E-03*	6.49E-02
ANN	GSSF	2.21E-10**	1.24E-07**	7.87E-03*	0.278 0.038	9.84E-01	3.66E-10**	4.90E-08**	1.60E-07**
	GSSS	2.20E-10**	9.99E-08**	8.43E-03*	8.62E-01	0.279 0.038	2.97E-12**	2.22E-08**	1.53E-08**
	P+13	3.26E-01	2.01E-01	3.91E-05**	1.07E-05**	1.16E-05**	**0.660** 0.027	6.85E-03*	2.30E-05**
	P+25	1.28E-02*	3.01E-02*	8.99E-01	2.06E-01	2.12E-01	1.86E-02*	0.572 0.047	1.32E-01
	ALL	5.47E-05**	1.55E-04**	2.31E-01	2.72E-03*	2.87E-03*	6.31E-05**	3.74E-01	0.524 0.055
	PSBC	7.04E-04**							
	THER		1.45E-11**						
	NG25			2.21E-12**					
GLM	GSSF				4.65E-06**				
	GSSS					4.37E-08**			
	P+13						3.23E-07**		
	P+25							4.99E-15**	
	ALL								1.68E-11**
	PSBC	3.20E-01							
	THER		2.36E-01						
	NG25			2.59E-04**					
SVM	GSSF				5.99E-02				
	GSSS					8.81E-02			
	P+13						1.35E-02*		
	P+25	2.42E-04**						4.71E-03*	
	ALL								6.29E-05**

GLM							
PSBC	THER	NG25	GSSF	GSSS	P+13	P+25	ALL
1.23E-01							
	8.68E-02						
		5.87E-03*					
			1.34E-03*				
				1.30E-02*			
1.71E-02*					1.54E-06**		
						3.96E-04**	
							1.12E-02*
**0.607 0.031**	9.42E-04**	3.83E-07**	1.44E-10**	1.21E-10**	6.56E-05**	8.09E-06**	9.71E-06**
1.38E-11**	0.511 0.844	1.33E-04**	1.75E-09**	5.22E-09**	2.08E-01	1.39E-02*	3.35E-02*
4.38E-12**	6.90E-03*	0.357 0.929	1.39E-05**	1.86E-04**	1.96E-03*	4.67E-02*	1.48E-02*
1.95E-06**	3.30E-13**	5.06E-14**	0.152 0.115	1.59E-01	1.05E-08**	1.59E-07**	4.77E-08**
2.28E-09**	1.23E-11**	3.28E-12**	1.75E-02*	0.201 0.091	4.71E-08**	1.22E-06**	2.83E-07**
3.92E-07**	2.31E-14**	1.51E-15**	6.80E-06**	4.08E-06**	0.474 0.257	1.61E-01	3.37E-01
7.73E-13**	1.79E-08**	3.43E-06**	3.77E-15**	4.65E-13**	2.20E-16**	0.439 1.109	6.17E-01
1.79E-11**	4.49E-11**	8.81E-11**	1.40E-11**	2.05E-11**	3.57E-12**	3.21E-10**	0.444 2.529
1.58E-04**							
	1.42E-11**						
		4.65E-12**					
			5.65E-06**				
				4.52E-08**			
					2.88E-07**		
2.24E-06**						7.93E-13**	
							1.77E-11**

SVM							
PSBC	THER	NG25	GSSF	GSSS	P+13	P+25	ALL
6.58E-01							
	3.28E-01						
		1.50E-01					
			8.51E-02				
				7.33E-01			
					2.89E-01	9.06E-03*	
						1.50E-04**	
							5.95E-05**
5.73E-02						1.57E-05**	
	1.35E-02*						
		1.16E-04**					
			9.55E-02				
				1.78E-02*			
					3.69E-07**		
						5.45E-08**	
							9.56E-07**
0.643 0.024	2.90E-03*	1.07E-05**	3.09E-09**	2.25E-13**	5.54E-02	4.05E-04**	9.51E-01
5.20E-03*	0.579 0.027	7.74E-03*	7.38E-09**	1.51E-11**	1.01E-04**	1.68E-06**	2.72E-03*
2.09E-05**	2.28E-02*	0.509 0.030	8.41E-08**	6.54E-09**	7.10E-07**	6.74E-08**	9.91E-06**
4.36E-12**	1.47E-08**	9.34E-07**	0.215 0.039	8.25E-02	3.31E-10**	6.30E-10**	2.71E-09**
4.68E-12**	2.47E-08**	3.46E-06**	7.11E-02	0.271 0.038	8.35E-14**	1.56E-14**	2.06E-13**
8.34E-02	3.18E-04**	2.50E-06**	3.97E-10**	2.42E-10**	0.681 0.022	1.07E-01	6.36E-02
1.16E-03*	5.20E-06**	7.78E-08**	2.66E-13**	1.41E-13**	1.86E-01	**0.711 0.020**	5.24E-04**
1.57E-01	5.08E-02	1.71E-04**	7.15E-13**	2.37E-12**	6.70E-03*	2.85E-05**	0.644 0.025

Diagonal cells from upper left to lower right contain the mean correlations *R* (upper) and *MSE* (lower) from the 10-fold cross validation predictions within the learning technique and mapping method, equivalent to the 10-fold cross validation *R* and *MSE* columns in table 2.

Cells above and to the right of the diagonal are the *t*-test probabilities of the 10-fold cross validations *R* rejecting the H_0_: x_a_ = x_b_, where x_a_ is mean *R* of combined technique and method a and x_b_ is the mean *R* of combined technique and method b.

Cells below and to the left of the diagonal are the *t*-test probabilities of the 10-fold cross validations *MSE* rejecting the H_0_: x_a_ = x_b_, where x_a_ is mean *MSE* of combined technique and method a and x_b_ is the mean *MSE* of combined technique and method b.

The cells off the upper left to lower right diagonal are unlabeled where P≥0.05.

The cells off the diagonal are labeled with a * where P<0.05 and P≥0.001 (<5.0E-02 and >1.0E-03).

The cells off the diagonal are labeled with a ** where P<0.001 or 1.0E-03.

Learning technique (TEC) and mapping method (MET) labels are consistent with Table 2.

**Figure 2:**
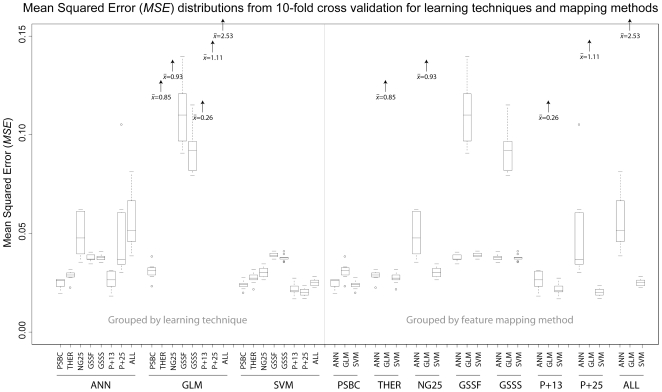
Box-and-whisker diagrams for the cross validation estimates of model accuracy performance, or Mean Squared Error (*MSE*). See Figure 1 for more details.

Similar to the comparisons of precision by comparing the means for *R* from cross validation replicates, the same comparisons can be made for the estimates for model accuracy, among the *MSE*s. The distribution ranges of the 10-fold cross validation estimates of *MSE* are presented in Figure 2, first grouped by learning technique, then by feature mapping method. It is again apparent in Figure 2 that the variances of the best performing method, PSBC, overlaps with the next most precise method, P+13 (*MSE*  = 0.027). For the ANN technique, the most accurate method, PSBC *MSE*  = 0.025, is not able to reject the null hypothesis of equality in the case of method P+13 *MSE*  = 0.027, *P* = 3.26E-01. Again, in the case of the comparisons between the method of PSBC and other methods within the ANN technique the null hypothesis of equality of *MSE* means, are able to be rejected with various degrees of statistical confidence, Table 7.

Within the GLM technique, the mapping method that results in the most precise model is the PSBC, *R* = 0.607, by 10-fold cross validation replicates. It is striking to note that this method dramatically outperforms other methods within the GLM technique, based on the lack of overlap in *R* estimate distributions, Figure 1. Consistent with the visual isolation of the PSBC among the other methods used to build models under the GLM technique, the *t*-test comparisons between the PSBC and the other methods all result in statistically significant rejection of the null hypotheses of equality of *R*. Even more striking in the GLM technique is that the PSBC method is the only technique that results in predictions of model accuracy (*MSE*) that are comparable with the other learning techniques, Figure 2. Some of the additional methods within the GLM technique result in dramatic inflation of the *MSE*, so while the precision of the models might be comparable, the model accuracies suffer. Statistical tests of the PSBC method clearly reject the null hypotheses of equality of the *MSE*s for other methods within the GLM technique.

Within the SVM learning technique, the method that produces the highest precision model is the P+25 method, *R* = 0.711. The distribution of P+25 method estimates of *R* only substantially overlap with the distribution of the P+13 method, Figure 1. Further statistical tests also suggest that the P+25 method outperforms all but the P+13 method for model precision, Table 7. For model accuracies, the SVM technique appears to provide uniformly smaller distributions of model *MSE*s, Figure 2. The most accurate method from within the SVM technique was also the P+25 method, *MSE*  = 0.020, but there is overlap between the *MSE* distributions between the P+25 and P+13 methods, Figure 2. Statistical tests reject the null hypotheses of equality between the P+25 method and the other methods, except for the P+13 method, Table 7.

#### IVb. within feature mapping method, between learning technique comparisons

General comparisons among learning techniques, but within a mapping method will provide a glimpse of how learning techniques might yield more or less effective models with the same group of features. The right hand portions of Figures 1 and 2 provide this visual glimpse between the 8 feature mapping methods, but focusing on the learning technique as the factor under study.

The PSBC method provides a uniformly high magnitude of *R* with low variance, Figure 1, and a low magnitude of *MSE* with low variance, Figure 2, across all learning techniques. Statistically, it is not possible to reject the different learning technique's abilities to build precise (as measured by *R*) models with the PSBC method, Table 7. Similarly, the accuracies of the models built with the PSBC method are not able to be discriminated between the ANN and SVM techniques, but are able to suggest a higher accuracy (lower *MSE*s) of both the ANN and SVM techniques when compared to the GLM technique, Table 7.

In general within a feature mapping method the ANN and SVM techniques always outperforms the GLM technique for precision (higher *R*s) and accuracy (lower *MSE*s) with various degrees of statistical significance, Table 7. Between ANN and SVM techniques the SVM provides a higher precision (higher *R*s) models in 6 of the 8 methods, with 2 of those 6 reaching statistical significance, Table 7. Between ANN and SVM techniques the SVM provides a higher accuracy (lower *MSE*s) in 8 of the 8 methods, with 4 of those 8 reaching various degrees of statistical significance, Table 7.

#### IVc. within best methods, between learning technique comparisons

It is apparent that various learning techniques have variable performance for building precise and accurate models under different feature mapping methods, but one objective of building predictive models is finding the modeling methods that result in the best model outcome. Among the learning methods, the most precise (highest *R*) models built under the ANN technique utilizes the P+13 method, *R* = 0.660. Similarly, the most precise model built under the GLM technique utilizes the PSBC method, *R* = 0.607, and the most precise model built under the SVM technique utilizes the P+25 method, *R* = 0.711. Statistically, the best-method SVM technique is able to reject the null hypotheses of equivalence between the best-method ANN technique, *P* = 9.06E-03, as well as the best-method GLM technique, *P* = 1.57E-05. Similarly, the best-method ANN technique is able to reject the null hypotheses of equivalence between the best-method GLM technique, *P* = 1.71E-02, Table 7.

Among the learning methods, the most accurate (lowest *MSE*) models built under the ANN technique utilizes the PSBC method, *MSE*  = 0.025. Similarly, the most accurate model built under the GLM technique utilizes the PSBC method, *MSE*  = 0.031, and the most accurate model built under the SVM technique utilizes the P+25 method, *R* = 0.020. Much like in comparisons of model precision, the best-method SVM technique is able to reject the null hypotheses of equivalence between the best-method ANN technique, *P* = 2.42E-04, as well as the best-method GLM technique, *P* = 2.24E-05. Similarly, the best-method ANN technique is able to reject the null hypotheses of equivalence between the best-method GLM technique, *P* = 7.04E-04, Table 7.

### V. Model combinations

There are several approaches that rely on more than a single model to make more informed decisions. Algorithms that apply bagging, boosting, stacking or other error correction methods can improve model performance by taking the strengths of some models to correct for other model weaknesses. To determine whether ANN, GLM and SVM learning technique models generate independent errors in their predictions, the ALL feature mapping method was used to train a model under each of the learning techniques. These models were then used to learn then predict the same dataset by parallel 10-fold cross validation. The residuals (residual  =  observed – predicted) for each of the 2431 data points was calculated for the ANN, GLM and SVM techniques. The residuals between models are all highly correlated between techniques (ANN-GLM *R* = 0.99, ANN-SVM *R* = 0.99, GLM-SVM *R* = 0.98). Model errors between learning techniques are apparently highly correlated, suggesting that these 3 models fail in a similar fashion and would not be suitable candidates for algorithms that systematically combine models to reduce error.

## Discussion

There are several optimality criteria that have been used in choosing between models and model construction systems. In no particular order, it is generally considered to be an improvement to: i) reduce the time of model construction, ii) reduce the complexity in implementing the method, iii) reduce the relative number of model parameters, iv) increase the exposure of the individual parameter contribution to the model for interpretation, v) increase the predictive precision of the model and vi) increase the predictive accuracy of the model. Previous comparisons among learning techniques and feature mapping methods for siRNAs have not generally used specific statistical methods to discriminate among the myriad of possible combinations. Here we suggest the use of and provide a demonstration of statistical models that maximizes both predictive model precision and accuracy that can discriminate among the high dimensionality of model space. Furthermore, from the observations here, it may be difficult to generalize the contributions of specific features when comparing among learning techniques as there are significant interactions among learning technique and feature that contribute to model performance. Stated plainly, the optimal feature set for maximizing the performance of a GLM model won't likely be the same feature set in an ANN or SVM model, or vice versa, therefore the learning technique influences what features are “relevant” in the model. Inferring “biological relevance” from “model relevance” when modeling technique has an influence on the features in the model is then questionable. Furthermore, any preference for model interpretability and the selection of a GLM based model may be somewhat self fulfilling where GLMs tend to perform best (among other GLMs, but not globally best) with a smaller number of features when compared to ANN or SVM models.

Overall, multiple tests are presented in Table 7 and the *P* values are not corrected for multiple tests. However, there are 28 planned comparisons within a single learning technique between the 8 presented methods, each among the measures of both precision and accuracy. If a Bonferroni correction is warranted as a way to adjust the type-I and type-II error rates, the typically used *P* value of 0.05 for the type-I error rate becomes 0.05/28 = 1.79E-03, and the cells in Table 7 labeled with ‘**’ still exceed this threshold. Additionally, 10-fold cross validation was used to generate the multiple replicate estimates of the model performance, but in cases where additional power is required for comparisons among models a higher order cross validation can be performed to increase the replication level and associated power of statistical tests.

It has been shown that the paired *t*-test is more liberal than the McNemar's test for classification learning problems [39], but the models tested here are regression models resulting in continuously distributed values and the tests presented in Table 7 are based on a 2 population *t*-tests without the assumption of homoscedasticity of population variances and using Welch's correction for degrees of freedom. For comparative purposes, the McNemar's test on these results can be found in supplementary materials (Table S1), and consistent with being a more liberal test the McNemar's test fails to reject the null hypothesis of equality for 24 *R* and 12 *MSE* comparisons while the 2 population *t*-test fails to reject 26 *R* and 17 *MSE* comparisons. The 2 population *t*-test is therefore a more conservative test than McNemar's, and more appropriate for the continuously distributed values that result from regression rather than classification procedures.

It is ill advised to use measures of model precision and accuracy that result from both training and testing on the same dataset. However, for comparative purposes these values are presented in this study, Table 8. Also, the use of a single kind of cross validation to reduce the problem of over-training models has not been universally adopted. The comparison of the present approaches to previously described methods for training and testing regression learning techniques for the same siRNA dataset are summarized in Table 8. It should be pointed out that many of the methods summarized in Table 8 are not being compared on an equal footing as their training sets were different or the dataset used in model testing was not available, but this is simply a proposed mechanism for making comparisons among predictive models when publishing the method. A complete comparison among techniques and methods is difficult due to the lack of many complementary metrics, the lack of availability of the algorithm's implementation or both. Adopting a common set of standard metrics for model comparison might allow ongoing refinements to be placed in a historical context or comparisons among approaches to take place in a quantitative fashion. A final proposal to allow extensible comparisons among a growing constellation of models would be to publish the individual replicates from any cross-validation procedure, as standard population level measures and comparisons such as *t*-tests (or other appropriate tests) would be possible across models, when published separately.

**Table 8:** Comparisons among regression learning techniques results for model precision and accuracy.

entire dataset	cross validation						
*R*	*MSE*	type	*R*	*MSE*	technique	availability	source	ref	Avail ability Dataset 2431
−0.513	-	-	-	-	Rule	-	Reynolds-Khvorova	[8]	-
−0.236	-	-	-	-	Rule	-	Uitei-Saigo v1	[9]	-
−0.457	-	-	-	-	Rule	-	Uitei-Saigo v2	[9]	-
−0.423	-	-	-	-	DT	-	Jagla-Rothman	[21]	-
−0.476	-	-	-	-	Boosting	-	Sætrom	[15]	-
−0.425	-	-	-	-	Rule	-	Amarzguioui-Prydz	[10]	-
−0.449	-	-	-	-	Rule	webserver(http://cluster-1.mpi-cbg.de/Deqor/deqor.html)	Henschel-Habermann	[17]	-
−0.666	-	-	-	-	ANN	Contact authors	Shabalina-Ogurtsov	[25]	-
−0.670	-	hold out	0.660	-	ANN	webserver(http://www.biopredsi.org)	Huesken-Hall	[18], [19]	+
−0.666	-	-	-	-	GLM	webserver(http://cbio.ensmp.fr/dsir)	Vert-Vandenbrouck	[27]	+
-	-	10-fold	-	92.3%^2^	SVM	webserver (http://optirna.unl.edu)	Ladunga	[30]	+
0.635	-	Single hold out	0.577	-	GLM	VB code(http://nar.oxforjournals.org/cgi/content/full/gkm699/DC1)	Ichihara	[31]	+
0.797	0.015	10-fold	0.760	0.023	SVM	C++ code(http://sourceforge.net/cprojects/seq2svm)	Peek	[32]	+
−0.578	-	-	-	-	GLM	data(http://gesteland.genetics.utah.edu/cmembers/olgaM/siRNA_database_cSeptember_2006.xls)	Matveeva model 1	[33]	+
−0.650	-	-	-	-	GLM	As above	Matveeva model 2	[33]	+
0.917	6.804^1^	-	-	-	SVM	webserver(http://www.bioinf.seu.edu.cn/csiRNA/index.html)	Jiang	[35]	+
0.726^3^	-	-	-	-	GLM	webserver(http://rna.chem.t.u-tokyo.ac.jp/csiexplorer.htm)	Katoh	[36]	+^3^
0.703	0.021	10-fold	0.636	0.025	ANN	C++ code(http://sourceforge.net/projects/cseq2svm)	Table 2	-	+
0.631	0.029	10-fold	0.607	0.031	GLM	As above	Table 2	-	+
0.931	0.006	10-fold	0.711	0.020	SVM	As above	Table 2	-	+

All values of *R* presented as negative values are from [33] as such a negative *R* model would not yield useful *MSE* values.

1) *MSE*, labeled as *RMSE*.

2) accuracy was defined as “100 minus the average percentage difference between predicted and observed knockdown activities”.

3) a dataset of 702 siRNAs was used, not the 2431 dataset considered by the remainder of the table.

Many of the conclusions here depend on the procedure of cross validation, and several kinds of cross validation have been suggested [39], [40], including 5×2-fold and 10×10-fold, as well as the 1×10-fold stratified method performed here. To help determine whether the choice of procedure for cross validation unduly influences the present results, the PSBC method was used to compare the mean and standard deviations resulting from various kinds of cross validation procedures across the ANN, GLM and SVM techniques, Table 9. In general, lower fold (2-fold, 3-fold) cross validation procedures tend to provide lower estimates of the *R* and higher estimates of the *MSE* due to their relatively smaller sizes of training sets when compared to the higher fold (10-fold, 20-fold) partitions. Also, there are some improvements seen in the reduction of the standard deviations by increasing fold partitions to 5, 10 and 20-fold, but there appears to be marginal benefit, from an estimation of the generalization error perspective, in progressing past 10-fold. Finally, 10 replicates of 10-fold (10×10-fold) and stratified 10-fold (1×10-fold) appear to have similar properties resulting in similar measures of central tendency and dispersion, and the 10-fold increased computational cost in the 10×10-fold might then be difficult to justify where learning algorithms are time intensive.

**Table 9:** Comparison of model cross-validation procedures on the PSBC feature mapping method across 3 learning techniques.

			ANN	ANN	GLM	GLM	SVM	SVM
Rep	Part	CV-fold	*R* (sd)	*MSE* (sd)	*R* (sd)	*MSE* (sd)	*R* (sd)	*MSE* (sd)
1	Strat	2	0.620 (2.09E-03)	0.0253 (5.47E-04)	0.586 (2.52E-02)	0.0334 (3.52E-03)	0.622 (7.44E-03)	0.0249 (4.73E-04)
1	Strat	3	0.625 (2.05E-02)	0.0249 (1.00E-03)	0.600 (2.14E-02)	0.0320 (2.13E-03)	0.626 (1.89E-02)	0.0247 (8.31E-04)
1	Strat	5	0.632 (3.19E-02)	0.0247 (2.16E-03)	0.600 (4.07E-02)	0.0315 (3.74E-03)	0.639 (3.46E-02)	0.0240 (1.86E-03)
**1**	**Strat**	**10**	**0.636 (3.63E-02)**	**0.0252 (2.78E-03)**	**0.607 (4.32E-02)**	**0.0309 (3.84E-03)**	**0.643 (3.56E-02)**	**0.0238 (2.05E-03)**
1	Strat	20	0.638 (5.00E-02)	0.0248 (2.85E-03)	0.611 (5.84E-02)	0.0307 (4.79E-03)	0.647 (4.85E-02)	0.0237 (2.71E-03)
1	Rand	2	0.616 (1.70E-02)	0.0258 (5.21E-04)	0.594 (1.19E-02)	0.0326 (1.60E-03)	0.619 (1.40E-02)	0.0251 (9.55E-04)
1	Rand	3	0.630 (2.29E-03)	0.0245 (1.04E-03)	0.604 (1.52E-02)	0.0316 (1.59E-03)	0.639 (4.47E-03)	0.0241 (1.20E-03)
1	Rand	5	0.630 (1.86E-02)	0.0247 (1.85E-03)	0.606 (2.85E-02)	0.0311 (2.65E-03)	0.636 (1.79E-02)	0.0242 (2.01E-03)
1	Rand	10	0.633 (3.84E-02)	0.0244 (2.24E-03)	0.608 (4.31E-02)	0.0309 (3.02E-03)	0.643 (3.56E-02)	0.0238 (2.46E-03)
1	Rand	20	0.637 (4.64E-02)	0.0247 (3.47E-03)	0.609 (5.13E-02)	0.0307 (3.50E-03)	0.646 (4.15E-02)	0.0237 (3.21E-03)
5	Rand	2	0.622 (9.79E-03)	0.0258 (1.26E-03)	0.594 (1.50E-02)	0.0326 (1.91E-03)	0.625 (1.19E-02)	0.0248 (7.22E-04)
5	Rand	3	0.632 (1.61E-02)	0.0250 (1.57E-03)	0.601 (1.97E-02)	0.0317 (1.84E-03)	0.636 (1.77E-02)	0.0242 (1.14E-03)
5	Rand	5	0.634 (2.58E-02)	0.0252 (1.55E-03)	0.605 (2.24E-02)	0.0312 (1.69E-03)	0.638 (1.61E-02)	0.0241 (1.25E-03)
5	Rand	10	0.633 (3.11E-02)	0.0248 (1.81E-03)	0.607 (3.34E-02)	0.0309 (2.15E-03)	0.642 (2.87E-02)	0.0239 (1.98E-03)
5	Rand	20	0.636 (5.12E-02)	0.0249 (3.40E-03)	0.608 (5.00E-02)	0.0308 (3.57E-03)	0.642 (4.82E-02)	0.0238 (3.36E-03)
10	Rand	2	0.622 (8.93E-03)	0.0256 (8.99E-04)	0.592 (1.35E-02)	0.0328 (1.69E-03)	0.625 (1.16E-02)	0.0248 (7.67E-04)
10	Rand	3	0.632 (1.33E-02)	0.251 (1.13E-03)	0.601 (1.68E-02)	0.0316 (1.64E-03)	0.636 (1.40E-02)	0.0242 (9.80E-04)
10	Rand	5	0.633 (2.46E-02)	0.0249 (1.91E-03)	0.606 (2.03E-02)	0.312 (1.67E-03)	0.638 (1.73E-02)	0.0241 (1.54E-03)
10	Rand	10	0.633 (3.59E-02)	0.0248 (2.06E-03)	0.608 (3.03E-02)	0.0309 (2.30E-03)	0.643 (2.78E-02)	0.0239 (2.13E-03)
10	Rand	20	0.636 (4.55E-02)	0.0249 (3.68E-03)	0.610 (4.63E-02)	0.0307 (3.77E-03)	0.644 (4.45E-02)	0.0238 (3.27E-03)
20	Rand	2	0.626 (1.18E-02)	0.0256 (1.18E-03)	0.593 (1.39E-02)	0.0327 (1.70E-03)	0.626 (1.19E-02)	0.0248 (7.08E-04)
20	Rand	3	0.630 (1.48E-02)	0.0250 (1.11E-03)	0.602 (1.67E-02)	0.0316 (1.65E-03)	0.636 (1.40E-02)	0.0242 (9.37E-04)
20	Rand	5	0.633 (2.54E-02)	0.0250 (1.47E-03)	0.606 (2.12E-02)	0.0311 (1.66E-03)	0.640 (1.90E-02)	0.0240 (1.38E-03)
20	Rand	10	0.634 (3.46E-02)	0.0249 (2.53E-03)	0.608 (3.24E-02)	0.0308 (2.46E-03)	0.644 (2.88E-02)	0.0238 (2.06E-03)
20	Rand	20	0.634 (5.05E-02)	0.0250 (3.36E-03)	0.609 (4.96E-02)	0.0307 (3.85E-03)	0.645 (4.58E-02)	0.0238 (3.22E-03)

Rep: replication level; Part: partitioning type, either stratification or random; CV-fold: cross-validation fold level; **Bold**: is the model cross validation procedure of single replicate stratified 10-fold cross validation.

From ANOVA results, measures of model precision can be explained rather well by a simple linear combination of (*R* model 3: *R* =  technique + method + error), with some evidence for interactions between techniques and methods contributing to the variance in *R*. By contrast, measures of model accuracy cannot be explained by a simple linear combination of technique and method, the model of that takes interactions between technique and method into account (*MSE* model 4: *MSE*  =  technique + method + (technique×method) + error) has a significantly better descriptive fit for the data. These observations suggest that finding highly precise models might simply be a matter of performing a 3-step process. The first step would be surveying learning techniques and choosing the technique with the greatest precision. The second step would involve surveying feature mapping methods and choosing the method, or feature set, with the greatest precision. The final step would combine the highly precise learning technique with the highly precise mapping method for the most precise model. By contrast, this 3-step process would not be suitable to finding highly accurate models, due to the large interaction component between technique and method seen in contributing to the variance in model accuracies (*MSE*). Finally, to address whether any one technique or method had excessive influence on the ANOVA results, each of the 3 techniques and 5 methods were sequentially removed and the ANOVA repeated (see supplementary materials Text S1 for regression CV data, Text S2 for R statistical analysis script on regression CV and Text S3 for results from R analysis on regression, similarly see Text S4, Text S5, and Text S6 for mean squared error CV data), and similar conclusions concerning variance partitions can be made under the leave one out analyses as with the entire dataset.

The degree of variability among learning techniques and feature mapping methods for measures of both model precision and accuracy are not equivalent. Overall for measures of precision, the learning techniques generally perform equally, but there are trends that suggest SVM techniques are more robust to the presence of noisy methods (features) than ANN and GLM techniques when adding other features. These observations would be consistent with SVM techniques tending to result in large numbers of features for robust models while ANN and GLM techniques would not be robust under those larger feature set scenarios, but would instead be better suited to smaller numbers of features that contain less noise.

By contrast, for measures of accuracy, there appear to be vast differences in learning techniques. For accuracy measures, SVM techniques tend to provide lower variance and smaller magnitude of errors. ANN techniques tend to provide small magnitudes of errors, but some feature methods appear to result in higher variability of accuracy measures. Finally, the GLM techniques tend to provide low accuracy models, where errors appear to be additive with the accumulation of more noisy features. The single exception to this low accuracy in GLM is for the method of PSBC, which is comparable to, but significantly under performs, the accuracies seen in the ANN and SVM techniques for this method. It is unclear to what degree one desirable property of GLM techniques outweighs ANN and SVM techniques in measures of precision and accuracy. Namely the explicit contribution of each feature to the final model in GLMs can be useful, but if model predictive precision and accuracy are quantifiable and lower than other techniques like ANN and SVM then model transparency will need to be given a higher priority than precision or accuracy in determining a desirable learning technique.

There are several limitations to the present study. First, the available siRNA data for constructing predictive models is limited. While the dataset under study is rather large, there are few additional siRNAs that have complete complementarity to their target mRNAs. So while there are near 600 additional 21-mer siRNAs with empirically measured activities [33], only 223 of these have complete complementarity to their respective target sequences due to a constant terminal dinucleotide DNA sequence “TT” in the siRNA's 3′ most positions, irrespective of whether the target mRNA possessed an “AA” sequence or not.

Second, it has been suggested that there is a positive association between a siRNA's activity and the physical location of the siRNA's target location in the mRNA [25]. Therefore when creating cross validation partitions for siRNAs, keeping siRNAs that share the same target footprint as siRNAs in the testing set would result in an upwards bias in estimates for precision and accuracy for that model. To investigate this possible source of bias, we implemented a cross-validation system that removed siRNAs from the training set that shared a target mRNA footprint with any siRNA in the testing set. In the stratified cross validation scheme with the SVM technique and P+25 feature mapping resulted in model *R* = 0.711 and *MSE*  = 0.020 with an average number of siRNAs in the training sets of 2187.9, Table 2. Cross validation that removed siRNAs from the training partition which share a footprint with any siRNA in the testing partition resulted in a model with an average among partitions of *R* = 0.694 and *MSE*  = 0.021 and an average number of siRNAs in the training sets of 2009.6. There is no significant difference between model precision (*R*: *t*-test, *P* = 0.310) or accuracy (*MSE*: *t*-test, *P* = 0.324) when excluding siRNAs from the training set that overlap with any of those in the testing set. These model comparison values result from testing on all 2431 siRNAs, across all partitions, but simply not all of the siRNAs are used to train the underlying model. So while there may be significant variance components in siRNA activity associated with the siRNA's target, these appear to have no statistically significant influence on the outcomes of predictive models when removing overlapping siRNAs from training partitions, or at least not specifically to the SVM technique applied to the P+25 feature method. The reduction of predictive power seen in removing siRNAs from the training set that overlap with the testing set is similar to the reduction of power seen in removing siRNAs in general from the training set, similar to the lower order folds in Table 9, not surprisingly reducing training data set size reduces model performance.

Third, the degree to which learning technique parameter tuning, additional features or feature selection methods results in the production of predictive models is not known. To place the learning techniques on a more even playing field, the parameters were optimized using the PSBC feature set, but it is likely that other optimal parameters could be found in the scenarios of additional or other features. A combinatory examination of 216 SVM parameter sets across the 8 feature methods (Table S2) suggests that first, not unpredictably, it is possible to de-tune effective parameters and produce less effective SVM models and second, the same general parameters optimized under the PSBC feature set produce maximally (or nearly so) predictive models under other feature sets. In general, it is possible to de-tune an ANN or SVM by choosing suboptimal model parameters to perform more poorly on the same feature set. Additional features, for example target secondary structures have been shown to be a significant factor [25], [27], [30], [32]–[34] in siRNA activity, and that feature set was not explored here, however adding target mRNA secondary structure features does not necessarily result in improved measures for model precision or accuracy if other features already dominate the model [32]. There were 279 distinct feature set combinations across 3 learning techniques for a total of 837 distinct models, but this is beyond doubt not an exhaustive exploration of model, parameter or feature space.

Certainly other sources of bias and error exist in the present study, but the intention here is to help determine to what degree the choice of machine learning technique and feature mapping method might produce different results in modeling siRNA effectiveness, possibly accounting for some of the heterogeneity seen in previously published studies modeling siRNA activity and what features produce maximally predictive models. These features have then been interpreted as the most relevant, but this interpretation needs to be placed clearly in the light of their relevance to a model's predictability and not necessarily of their biological relevance. The methods and techniques presented here are all available for download from sourceforge.net (http://sourceforge.net/projects/seq2svm/) as a group of C++ classes and interfaces for their execution. Finally, to provide access to additional data mining and learning techniques in a graphical interface, there is also an executable that transforms a siRNA dataset, by various methods, into an attribute-relation file format (ARFF), appropriate for use in the Waikato environment for knowledge analysis (WEKA).

## Materials and Methods

### Learning Techniques

Three learning techniques were investigated. The first was artificial neural networks (ANN), as implemented in the FANN C++ library (http://leenissen.dk/fann/). The second was a general linear model (GLM), as implemented in the Numerical Recipes library. The last was a support vector machine (SVM), as implemented in the libsvm library (http://www.csie.ntu.edu.tw/~cjlin/libsvm/). Additional techniques for machine learning can be found as they are implemented at the WEKA package (http://www.cs.waikato.ac.nz/ml/weka/). Software that performs the presently described machine learning techniques and analytical methods can be found at Sourceforge.net (http://sourceforge.net/projects/seq2svm/). To clarify the language in the present manuscript the learning processes of ANN, GLM and SVM are referred to, as a group, as techniques.

### Feature Mapping Methods

Five general feature mapping methods were used in this study, given in the order of their previously determined ability to build predictive models:

(PSBC) position specific base composition. Method 1, previously described from [32].
(THER) thermodynamic parameters from an RNA nearest neighbor algorithm. Method 2, previously described from [32].
(NG25) *N*-Grams or motifs of length 2 through 5. Method 11, previously described from [32].
(GSSF) guide strand secondary structure-features, a combination of secondary structure and base composition of the guide strand proposed by Xue *et al*., [41]. Method 5, previously described from [32].
(GSSS) predicted guide strand secondary structure. Method 4, previously described from [32].

Nucleic acid secondary structures were predicted with Vienna RNA library (http://www.tbi.univie.ac.at/~ivo/RNA/). For more details in specific features see [32]. Feature mapping methods result in the production of features or feature sets, and for clarity these are referred to as the means for their production, or “methods” rather than as the noun resulting from their production, or “feature set”. However, these can be considered interchangeable and to maintain consistency with the noun learning “technique” we use the noun feature mapping “method”.

### Learning Technique optimization

Learning technique parameters were optimized by using a course grid search method on the ANN, GLM or SVM techniques by using the PSBC method alone to maximize the *R* fit under a 10-fold cross-validation scheme. Analysis of variance (ANOVA) and other statistical tests were performed with the R statistical package (http://www.r-project.org/).

### siRNA Data

The 2431 siRNAs of length 21 nucleotides with complete base pairing to their respective target sequences from a siRNA study were used as the empirical activity data to study [18], [19].

## Supporting Information

Table S1 McNemar's test of statistically significant differences among measures of model precision and accuracy
(0.37 MB DOC)

Table S2 cross validation grid search for optimized SVM parameters
(0.10 MB XLS)

Text S1 regression CV data
(0.00 MB TXT)

Text S2 Regression R input script
(0.26 MB TXT)

Text S3 Regression R output
(0.46 MB TXT)

Text S4 MSE CV data
(0.00 MB TXT)

Text S5 MSE R input script
(0.26 MB TXT)

Text S6 MSE R output
(0.45 MB TXT)
