# Supplementary material for: Comparing Artificial Neural Networks, General Linear Models and Support Vector Machines in Building Predictive Models for Small Interfering RNAs
Source: PLoS One. 2009 Oct 22;4(10):e7522. doi: 10.1371/journal.pone.0007522 (PMC2760777; doi:10.1371/journal.pone.0007522)
Supplement: Table S1 — McNemar's test of statistically significant differences among measures of model precision and accuracy (0.37 MB DOC) [file pone.0007522.s001.doc]

Supplementary Table McNemar’s test.

Comparison among learning technique and mapping method for building significantly dissimilar models by 10-fold cross validation with McNemar’s test.

|  |  |  |  |  |  |  |  |  |  |  |  |  |  |  |  |  |  |  |  |  |  |  |  |  |  |  |  |  |
| --- | --- | --- | --- | --- | --- | --- | --- | --- | --- | --- | --- | --- | --- | --- | --- | --- | --- | --- | --- | --- | --- | --- | --- | --- | --- | --- | --- | --- |
| TEC |  |  |  |  | ANN |  |  |  |  |  |  |  |  | GLM |  |  |  |  |  |  |  |  | SVM |  |  |  |  |  |
|  | MET | PSBC | THER | NG25 | GSSF | GSSS | P+13 | P+25 | ALL |  | PSBC | THER | NG25 | GSSF | GSSS | P+13 | P+25 | ALL |  | PSBC | THER | NG25 | GSSF | GSSS | P+13 | P+25 | ALL |  |
|  | PSBC | 0.636  **0.025** | 2.58E-03 | 1.48E-03 | 8.50E-04 | 8.50E-04 | 1.04E-01 | 1.46E-02 | 2.58E-03 |  | 5.14E-02 |  |  |  |  |  |  |  |  | 2.42E-01 |  |  |  |  |  |  |  |  |
|  | THER | 1.48E-03 | 0.567  0.029 | 6.44E-02 | 8.50E-04 | 8.50E-04 | 2.58E-03 | 2.70E-02 | 1.21E-01 |  |  | 1.21E-01 |  |  |  |  |  |  |  |  | 6.44E-01 |  |  |  |  |  |  |  |
|  | NG25 | 1.48E-03 | 2.58E-03 | 0.464  0.049 | 2.58E-03 | 1.48E-03 | 1.48E-03 | 1.46E-02 | 2.42E-01 |  |  |  | 2.70E-02 |  |  |  |  |  |  |  |  | 2.42E-01 |  |  |  |  |  |  |
| ANN | GSSF | 8.50E-04 | 8.50E-04 | 8.11E-03 | 0.278  0.038 | 2.42E-01 | 8.50E-04 | 1.48E-03 | 8.50E-04 |  |  |  |  | 2.70E-02 |  |  |  |  |  |  |  |  | 2.70E-02 |  |  |  |  |  |
|  | GSSS | 8.50E-04 | 8.50E-04 | 8.11E-03 | 1.04E-01 | 0.279  0.038 | 8.50E-04 | 1.48E-03 | 8.50E-04 |  |  |  |  |  | 2.70E-02 |  |  |  |  |  |  |  |  | 1.00E-00 |  |  |  |  |
|  | P+13 | 8.07E-01 | 2.70E-02 | 2.58E-03 | 8.50E-04 | 8.50E-04 | **0.660**  0.027 | 8.11E-03 | 4.55E-03 |  | 8.11E-03 |  |  |  |  | 1.48E-03 |  |  |  |  |  |  |  |  | 1.04E-01 | 1.46E-02 |  |  |
|  | P+25 | 1.46E-02 | 1.46E-02 | 3.50E-01 | 5.14E-02 | 5.14E-02 | 1.46E-02 | 0.572  0.047 | 3.54E-02 |  |  |  |  |  |  |  | 8.11E-03 |  |  |  |  |  |  |  |  | 1.48E-03 |  |  |
|  | ALL | 1.48E-03 | 2.58E-03 | 2.42E-01 | 8.11E-03 | 8.11E-03 | 1.48E-03 | 3.50E-01 | 0.524  0.055 |  |  |  |  |  |  |  |  | 2.70E-02 |  |  |  |  |  |  |  |  | 2.58E-03 |  |
|  |  |  |  |  |  |  |  |  |  |  |  |  |  |  |  |  |  |  |  |  |  |  |  |  |  |  |  |  |
|  | PSBC | 1.48E-03 |  |  |  |  |  |  |  |  | **0.607**  **0.031** | 4.55E-03 | 8.50E-04 | 8.50E-04 | 8.50E-04 | 2.58E-03 | 1.48E-03 | 2.58E-03 |  | 2.70E-02 |  |  |  |  |  | 2.58E-03 |  |  |
|  | THER |  | 8.50E-04 |  |  |  |  |  |  |  | 8.50E-04 | 0.511  0.844 | 4.55E-03 | 8.50E-04 | 8.50E-04 | 1.00E-00 | 2.70E-02 | 5.14E-02 |  |  | 2.70E-02 |  |  |  |  |  |  |  |
|  | NG25 |  |  | 8.50E-04 |  |  |  |  |  |  | 8.50E-04 | 1.46E-02 | 0.357  0.929 | 8.50E-04 | 1.46E-02 | 1.46E-02 | 5.14E-02 | 2.70E-02 |  |  |  | 4.55E-03 |  |  |  |  |  |  |
| GLM | GSSF |  |  |  | 8.50E-04 |  |  |  |  |  | 8.50E-04 | 8.50E-04 | 8.50E-04 | 0.152  0.115 | 2.42E-01 | 8.50E-04 | 8.50E-04 | 8.50E-04 |  |  |  |  | 1.04E-01 |  |  |  |  |  |
|  | GSSS |  |  |  |  | 8.50E-04 |  |  |  |  | 8.50E-04 | 8.50E-04 | 8.50E-04 | 1.46E-02 | 0.201  0.091 | 8.50E-04 | 1.48E-03 | 8.50E-04 |  |  |  |  |  | 1.46E-02 |  |  |  |  |
|  | P+13 |  |  |  |  |  | 8.50E-04 |  |  |  | 8.50E-04 | 8.50E-04 | 8.50E-04 | 2.58E-03 | 1.48E-03 | 0.474  0.257 | 5.14E-02 | 1.00E-00 |  |  |  |  |  |  | 1.48E-03 |  |  |  |
|  | P+25 |  |  |  |  |  |  | 8.50E-04 |  |  | 8.50E-04 | 8.50E-04 | 1.48E-03 | 8.50E-04 | 8.50E-04 | 8.50E-04 | 0.439  1.109 | 2.42E-01 |  |  |  |  |  |  |  | 8.50E-04 |  |  |
|  | ALL |  |  |  |  |  |  |  | 8.50E-04 |  | 8.50E-04 | 8.50E-04 | 8.50E-04 | 8.50E-04 | 8.50E-04 | 8.50E-04 | 8.50E-04 | 0.444  2.529 |  |  |  |  |  |  |  |  | 1.48E-03 |  |
|  |  |  |  |  |  |  |  |  |  |  |  |  |  |  |  |  |  |  |  |  |  |  |  |  |  |  |  |  |
|  | PSBC | 2.42E-01 |  |  |  |  |  |  |  |  | 2.58E-03 |  |  |  |  |  |  |  |  | 0.643  0.024 | 4.55E-03 | 2.58E-03 | 8.50E-04 | 8.50E-04 | 2.70E-02 | 8.11E-03 | 8.07E-01 |  |
|  | THER |  | 3.54E-02 |  |  |  |  |  |  |  |  | 8.50E-04 |  |  |  |  |  |  |  | 4.55E-03 | 0.579  0.027 | 1.04E-01 | 8.50E-04 | 8.50E-04 | 2.58E-03 | 1.48E-03 | 8.11E-03 |  |
|  | NG25 |  |  | 2.58E-03 |  |  |  |  |  |  |  |  | 8.50E-04 |  |  |  |  |  |  | 4.55E-03 | 1.04E-01 | 0.509  0.030 | 8.50E-04 | 8.50E-04 | 8.50E-04 | 8.50E-04 | 2.58E-03 |  |
| SVM | GSSF |  |  |  | 1.46E-02 |  |  |  |  |  |  |  |  | 8.50E-04 |  |  |  |  |  | 8.50E-04 | 8.50E-04 | 1.48E-03 | 0.215  0.039 | 1.46E-02 | 8.50E-04 | 8.50E-04 | 8.50E-04 |  |
|  | GSSS |  |  |  |  | 2.42E-01 |  |  |  |  |  |  |  |  | 8.50E-04 |  |  |  |  | 8.50E-04 | 8.50E-04 | 1.48E-03 | 1.46E-02 | 0.271  0.038 | 8.50E-04 | 8.50E-04 | 8.50E-04 |  |
|  | P+13 |  |  |  |  |  | 8.11E-03 |  |  |  |  |  |  |  |  | 8.50E-04 |  |  |  | 2.70E-02 | 2.58E-03 | 1.48E-03 | 8.50E-04 | 8.50E-04 | 0.681  0.022 | 1.04E-01 | 1.21E-01 |  |
|  | P+25 | 4.55E-03 |  |  |  |  |  | 1.48E-03 |  |  | 1.48E-03 |  |  |  |  |  | 8.50E-04 |  |  | 8.11E-03 | 1.48E-03 | 8.50E-04 | 8.50E-04 | 8.50E-04 | 1.04E-01 | **0.711**  **0.020** | 8.11E-03 |  |
|  | ALL |  |  |  |  |  |  |  | 1.48E-03 |  |  |  |  |  |  |  |  | 8.50E-04 |  | 3.50E-01 | 1.46E-02 | 4.55E-03 | 8.50E-04 | 8.50E-04 | 1.98E-02 | 4.55E-03 | 0.644  0.025 |  |
|  |  |  |  |  |  |  |  |  |  |  |  |  |  |  |  |  |  |  |  |  |  |  |  |  |  |  |  |  |

Diagonal cells from upper left to lower right contain the mean correlations *R* (upper) and *MSE* (lower) from the 10-fold cross validation predictions within the learning technique and mapping method, equivalent to the 10-fold cross validation *R* and *MSE* columns in table 2­.

Cells above and to the right of the diagonal are the *t*-test probabilities of the 10-fold cross validations *R* rejecting the H_0_: x_a_ = x_b_, where x_a_ is mean *R* of combined technique and method a and x_b_ is the mean *R* of combined technique and method b.

Cells below and to the left of the diagonal are the *t*-test probabilities of the 10-fold cross validations *MSE* rejecting the H_0_: x_a_ = x_b_, where x_a_ is mean *MSE* of combined technique and method a and x_b_ is the mean *MSE* of combined technique and method b.

The cells off the upper left to lower right diagonal are shaded blue where P ≥ 0.05.

The cells off the diagonal are shaded yellow where P < 0.05 and P ≥ 0.001 (< 5.0E-02 and > 1.0E-03).

The cells off the diagonal are shaded red where P < 0.001 or 1.0E-03.

Learning technique (TEC) and mapping method (MET) labels are consistent with Table 2.
